# Treating Temporomandibular Disorders Through Orthodontics: A Scoping Review of Evidence, Gaps, and Clinical Guidance

**DOI:** 10.3390/clinpract15100182

**Published:** 2025-09-30

**Authors:** Man Hung, Jacob Daniel Gardner, Samantha Lee, Wendy C. Birmingham, Richard M. Stevens, Connor Schwartz, Nader Karimi, Amir Mohajeri

**Affiliations:** 1 College of Dental Medicine DMD Program, Roseman University of Health Sciences, South Jordan, UT 84095, USA; 2 College of Dental Medicine AEODO Program, Roseman University of Health Sciences, Henderson, NV 89014, USA; 3 Department of Orthopaedic Surgery Operations, University of Utah, Salt Lake City, UT 84108, USA; 4 Division of Public Health, University of Utah, Salt Lake City, UT 84108, USA; 5 The Wharton School, University of Pennsylvania, Philadelphia, PA 19104, USA; 6 Utah Department of Veterans and Military Affairs, Salt Lake City, UT 84148, USA; 7 Department of Psychology, Brigham Young University, Provo, UT 84602, USA; 8 Library, Roseman University of Health Sciences, South Jordan, UT 84095, USA; 9 Library, Noorda College of Osteopathic Medicine, Provo, UT 84606, USA

**Keywords:** temporomandibular disorders, orthodontic treatment, orthognathic surgery, temporary anchorage devices, patient-centered outcomes

## Abstract

**Introduction:** Evidence on orthodontic interventions for temporomandibular disorders (TMD) is fragmented and inconclusive, creating a gap in guidance for clinical decision-making. This study addresses that gap by evaluating current knowledge on these interventions. **Methods:** A PRISMA-ScR scoping review was conducted with a systematic search of PubMed, Scopus, and Web of Science (2018–2023). Eligible studies were peer-reviewed, English-language, human studies examining TMD treatment and/or etiology. Three independent reviewers screened records and extracted data and a fourth reviewer performed random audits. **Results:** Of 899 records, 10 studies met inclusion criteria (non-surgical, *n* = 7: 4 case reports, 2 prospective, 1 longitudinal; combined orthodontic–surgical, *n* = 3: 1 case report, 2 longitudinal; participant ages 15–71 years). Diagnostics included imaging, clinical examination, occlusal analysis, and questionnaires, although few used RDC/TMD or DC/TMD criteria. Non-surgical orthodontic modalities (fixed appliances, camouflage, TADs, stabilization splints) showed mixed results, with several studies reporting short-term symptom improvement, while others found no effect on TMD onset or progression. Combined orthodontic–surgical approaches (e.g., bilateral sagittal split osteotomy, Le Fort I) also showed variable outcomes. **Conclusions:** Low-to-moderate quality evidence suggests that orthodontic-surgical interventions may alleviate TMD symptoms in select patients; however, heterogeneity and limited use of standardized diagnostics constrain the certainty of these findings. Future research should prioritize DC/TMD-based diagnostics, core outcomes, comparative designs, and ≥12–24 months of follow-up to identify prognostic factors and responsive subgroups.

## 1. Introduction

Temporomandibular disorders (TMD) are a heterogeneous class of musculoskeletal disorders that primarily affect the masticatory muscles, temporomandibular joint (TMJ) complex, and the surrounding bony structures. They encompass a spectrum of conditions, including myofascial pain, internal joint derangement, degenerative joint disease, and arthralgia, each of which can cause pain and dysfunction in the jaw joint and the muscles that control jaw movement. The TMJ is a critical structure that connects the lower jaw to the temporal bone of the skull, allowing the movements necessary for speaking, chewing, and other functions. TMDs represent the most common clinicopathologic conditions of the masticatory apparatus, with an estimated ten million individuals affected in the United States alone [1], with higher prevalence [2,3,4,5,6,7,8] and greater average symptom severity in women than in men.

The symptoms of TMD are diverse and can include jaw pain, jaw locking, joint noises, restricted mouth opening, masticatory fatigue, frequent severe headaches, tinnitus, vertigo, and impaired oral functions [2,9,10]. The pain associated with TMD can range from intermittent, activity-related discomfort to persistent, debilitating pain that limits daily activities. Additionally, individuals with TMD may experience headaches, earaches, and facial pain, symptoms that are often mistaken for other medical conditions. Other common symptoms include difficulty chewing, a clicking or popping sound in the jaw, and muscle spasms in the jaw or neck. The etiology of TMD remains incompletely understood; much of the historical clinical focus has been on occlusal disharmony (malocclusion) as a potential causal agent [11]. Additionally, TMD may also be related to several occlusal traits, such as open bite, deep bite, and posterior crossbite [8,12]. However, contemporary evidence supports a multifactorial model, involving interacting genetic, environmental, biomechanical, and behavioral factors, making it unlikely that a single cause explains all cases [13]. Proposed etiological factors include muscle hyperactivity, central pain mechanisms, injury or trauma [14], oral parafunctions (e.g., bruxism), genetic markers, and psychological distress including stress, depression, anxiety, and catastrophizing behaviors [11,15]. Individuals with more severe TMD often report a significantly lower quality of life [16], experiencing difficulties in performing routine activities and maintaining social relationships.

Over the past 15 years, longitudinal work from the Orofacial Pain: Prospective Evaluation and Risk Assessment (OPPERA) studies has anchored TMD within a modern biopsychosocial framework, identifying multiple interacting risk domains—peripheral and central nociceptive processing, psychological distress, sleep disturbance, injury, and genetic/biological factors—and documenting overlap with other chronic pain conditions [14,15]. Within this paradigm, dental occlusion is best considered a contextual biomechanical contributor rather than a singular cause, which cautions against over-attributing TMD onset or persistence to malocclusion alone. This framing also explains why interventions that modify occlusion or loading (e.g., orthodontics, orthognathic surgery) may relieve symptoms in selected patients while failing to produce uniform or durable benefits across heterogeneous populations.

Treatment for TMD varies depending on the severity and putative underlying cause. First-line, conservative approaches include physical therapy, behavioral interventions and stress management, oral orthotics/splints, and medications such as nonsteroidal anti-inflammatory drugs and muscle relaxants. In more severe or refractory cases, dental interventions, orthodontic treatments, or surgical procedures may be considered to correct structural abnormalities and alleviate symptoms. A consistently high proportion of patients presenting for orthodontic care report TMD complaints, and studies have demonstrated that patients with TMD who complete orthodontic treatment have shown improvements in oral function and symptom burden, such as better oral hygiene, less muscle tenderness, and improved sleep quality, compared to those who have not undergone orthodontic treatment [17,18]. Conversely, there are also studies that link the likelihood of developing TMD to orthodontic treatment [19], while other studies suggest no association between orthodontics and TMD development [20]. This heterogeneity of findings underscores the methodological variability across studies (e.g., case definitions, outcome measures, follow-up intervals) and highlights the need for further research to clarify and confirm the potential link between orthodontic interventions and TMD [21].

Additionally, there are significant differences in beliefs regarding best clinical management practices for TMD among orthodontists and other dental specialists [17]. Some research claims that orthodontic treatment can prevent or ameliorate TMD by achieving occlusal harmony or repositioning mandibular condyles to their optimal position in the glenoid fossae [22], while other research notes that traditional orthodontic treatments may neglect the concept of functional occlusion and could trigger the development of TMD [23]. Yet further research shows that orthodontic treatments to correct skeletal Class II or Class III malocclusions did not—during or after treatment—either increase or decrease the risk of developing TMD [17,24,25,26,27], nor did they reliably cause or prevent TMD [28], though targeted orthodontic care may help to alleviate TMD pain in selected patients [29]. Taken together, clear, cross-disciplinary guidance for TMD management remains limited, and the potential link between TMD and orthodontic treatment continues to be debated [30].

Given the lack of consensus and the inherent complexity of TMD [17], the present study evaluates the effectiveness of orthodontic interventions in patients with TMD, systematically assessing outcomes across pain intensity, jaw function, joint sounds, patient-reported quality of life, and adverse effects. To balance recency with conceptual rigor, we retained an inclusion window of 2018 to 2023 to capture contemporary diagnostics and modalities (e.g., DC/TMD usage, aligner-era mechanics, temporary anchorage devices, current orthognathic protocols), while explicitly situating interpretation within the established OPPERA framework. We aimed to map (rather than infer causality about) recent evidence on orthodontic and combined orthodontic–surgical interventions for TMD using PRISMA-ScR guidance [31], summarize outcomes across a core outcome set (pain, function, joint sounds, PROs, adverse events), evaluate diagnostic approaches (RDC/TMD or DC/TMD), and highlight evidence gaps and practice implications.

## 2. Methods

### 2.1. Protocol Reporting

This review followed the Preferred Reporting Items for Systematic Reviews and Meta-Analyses guidelines and, given the scoping nature of parts of our question, the PRISMA-ScR extension where applicable [31] to analyze literature related to TMD in orthodontic patients. The primary objective was to synthesize articles published within the last five years, focusing on human-based studies conducted in the United States (U.S.). The rationale for this focus on recent literature was to capture the most current evidence amidst evolving diagnostic techniques and treatment modalities in TMD research. The review specifically aimed to address the treatment methods and underlying causes of TMD in orthodontic care. A protocol specifying objectives, eligibility criteria, outcomes, and analysis plans was developed a priori.

### 2.2. Eligibility Criteria

We conducted a scoping review to map recent evidence on orthodontic interventions for TMD. We included peer-reviewed, human studies published between 1 January 2018 and 31 December 2023 that examined TMD treatment and/or etiology in an orthodontic context. Eligible designs reflected the breadth appropriate for a scoping review and comprised randomized controlled trials, quasi-experimental studies, cohort studies, case–control studies, cross-sectional studies, and case reports/series. Interventions encompassed non-surgical orthodontic modalities (e.g., fixed appliances/aligners, camouflage mechanics, miniscrews/temporary anchorage devices, stabilization splints used adjunctively) and combined orthodontic–orthognathic approaches (e.g., BSSO, Le Fort I with staged orthodontics). To ensure clinical relevance, studies had to report at least one TMD outcome within a core outcome set (pain intensity, jaw function/mouth opening, joint sounds) and/or patient-reported outcomes/quality of life or adverse events. Because diagnostic standardization is central to interpretability, we recorded whether studies used RDC/TMD or DC/TMD and their diagnostic methods (clinical examination, imaging, occlusal analysis, validated questionnaires); use of RDC/TMD/DC/TMD was not an inclusion requirement.

We excluded records without full-text availability; review articles (narrative, scoping, systematic, and meta-analyses), editorials/opinions; unpublished or retracted articles (including preprints); non-English publications; non-human studies; and studies that did not address TMD in relation to orthodontics (e.g., isolated TMJ surgery without orthodontic components; purely prosthodontic/occlusal adjustment without an orthodontic context). Full inclusion/exclusion criteria are summarized in Table 1, and reasons for full-text exclusions are presented in the PRISMA flow diagram. The 2018–2023 window was chosen to reflect contemporary diagnostics and treatment modalities; foundational work predating 2018 is cited in the Introduction to contextualize findings.

These criteria were established to ensure that the review concentrated on recent, high-quality studies on the relationship between TMD and orthodontics. Limiting the search to articles from the past five years ensured that the most current and relevant information was included, reflecting the latest advancements and trends in the field. Requiring peer-reviewed articles guaranteed that the selected studies adhered to rigorous research standards, ensuring reliability and validity of the findings. Incorporating only English-language articles maintained consistency and accuracy in interpretation, thereby avoiding potential translation errors that could compromise the integrity of the analysis. This also facilitated a clearer and more precise synthesis of the data. The focus on human-based studies was essential to ensure the direct applicability and relevance of the findings to the target population, thus enhancing the practical implications of the review. Furthermore, the deliberate exclusion of review articles aimed to avoid redundancy and promote the synthesis of new and original data, insights, and findings. This approach ensured that the review added unique value to the existing body of knowledge by highlighting novel research and perspectives, ultimately contributing to a more comprehensive and up-to-date understanding of the effectiveness of orthodontic interventions in managing TMD. For the “U.S.-based” restriction, we defined eligibility as studies in which the study setting and data collection occurred within the U.S; multinational studies were included only if U.S.-specific data were separately reportable. We intentionally restricted inclusion to U.S.-based studies to maximize applicability to U.S. orthodontic practice and reduce heterogeneity introduced by cross-country differences in care pathways (insurance/coverage, device availability and regulatory status, surgical protocols, and adoption of DC/TMD). The U.S.-only scope is a pre-specified boundary to enhance clinical relevance and interpretability within a single health system.

### 2.3. Information Sources and Search Strategy

To identify relevant articles for this review, we conducted comprehensive searches across multiple databases, including PubMed, Scopus, and Web of Science. The search terms employed were specifically chosen to capture the scope of the study. These terms included “orthodontics” or “orthodontics, corrective” (MeSH terms), “temporomandibular joint disorders” (MeSH), and “malocclusion” or “occlusion” (MeSH). These keywords were carefully selected to ensure the retrieval of studies pertinent to the treatment or causes of TMD in orthodontic patients. Searches were limited to human studies and English language; no country restriction was applied. A detailed representation of the specific search terms and databases utilized can be found in Supplementary Materials Table S1. This thorough search strategy was designed to encompass a wide range of literature relevant to the research objectives.

### 2.4. Selection Process

Records were de-duplicated and screened in two stages. Three reviewers independently screened titles and abstracts for eligibility based on the predefined inclusion and exclusion criteria. Reasons for exclusion were documented at this stage when applicable. Candidate records then underwent independent full-text review by the same three reviewers, who again documented explicit exclusion reasons (e.g., non-U.S. setting, review article, no orthodontic context, no TMD outcomes, unavailable full text). Disagreements at either stage were resolved by discussion and consensus. A fourth reviewer performed random audits of screening decisions to verify consistency with the protocol. The PRISMA flow diagram summarizes the numbers at each stage and the distribution of full-text exclusion reasons. Articles that passed the initial screening were subjected to a comprehensive full-text analysis. During this phase, key information was extracted, including sample size, age range, study design, types of TMD, diagnostic methods, the role of orthodontic treatment in TMD, and study conclusions. To ensure the accuracy and completeness of the extracted data, a secondary reviewer independently repeated the extraction process. This redundancy helped to confirm that all relevant information was captured and that any discrepancies were identified and resolved.

### 2.5. Data Collection Process

For all studies meeting eligibility, data were extracted using a standardized form that was piloted prior to use. One reviewer performed primary extraction and a second reviewer independently verified each record to ensure completeness and accuracy. Extracted items included: study design; setting (U.S.); sample size; age range (and sex, if reported); TMD subtype(s); diagnostic framework (RDC/TMD or DC/TMD) and diagnostic methods (clinical examination, imaging, occlusal analysis, validated questionnaires); orthodontic intervention category (non-surgical versus combined orthodontic–surgical); outcomes mapped to a core set (pain intensity, jaw function/mouth opening, joint sounds, patient-reported outcomes/quality of life, adverse events); and follow-up duration. Discrepancies between extractors were reconciled by consensus.

### 2.6. Critical Appraisal (Risk of Bias)

Consistent with scoping review methodology, studies were not excluded based on appraisal. We performed design-appropriate descriptive appraisal using JBI tools for case reports/series and cohorts (selection bias, confounding, measurement, follow-up). We summarize risk qualitatively in Results and Discussion.

### 2.7. Synthesis Methods

The synthesis of the data involved a thematic approach. This method aimed to systematically analyze and integrate findings from the included studies, focusing on identifying common themes, patterns, and trends. The goal was to gain a deeper understanding of the relationship between TMD and orthodontic treatment. By organizing the findings thematically, we could highlight significant insights and draw meaningful conclusions regarding the impact of orthodontic treatment on TMD. This approach not only provided a structured way to analyze complex data but also facilitated the identification of gaps in the current literature, suggesting areas for future research. By systematically integrating and analyzing the findings, we were able to provide a perspective on the interplay between orthodontic treatment and TMD, offering valuable insights for clinicians and researchers aiming to optimize treatment strategies for TMD patients.

### 2.8. Ethics and Funding

This review analyzed published data only and did not require institutional review board approval. No external funding influenced study selection, appraisal, or synthesis.

## 3. Results

### 3.1. Article Selection

**Figure 1:**
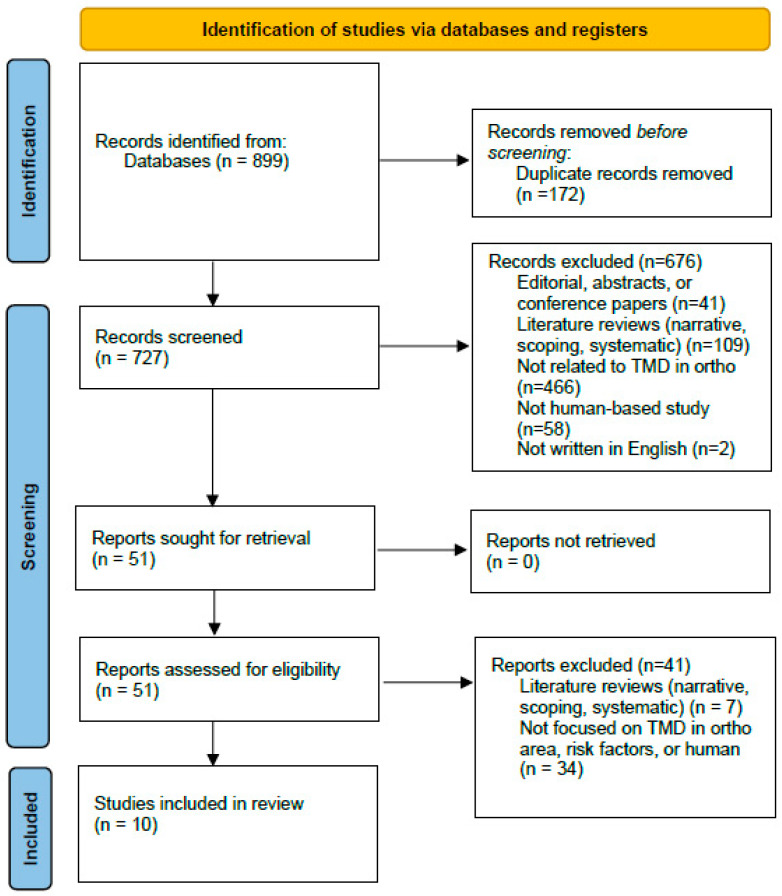
Flowchart for the literature selection process.

Figure 1 provides a detailed illustration of the article selection process employed in this study. Initially, a total of 899 articles were identified through the search. After removing duplicate entries, the dataset was reduced to 727 unique articles (172 duplicates; 19.1% of records). These 727 articles underwent a preliminary screening based on their titles and abstracts to determine their relevance and ensure they met the basic inclusion criteria established for the review. Following this initial screening, 51 articles were deemed relevant and were selected for a more detailed and thorough in-depth review (7.0% of screened titles/abstracts). During this evaluation phase, each article was carefully examined to assess its suitability and alignment with the specific objectives of the scoping review. This evaluation process ultimately led to the inclusion of 10 articles in the final scoping review (1.4% of unique records; 19.6% of full-texts assessed). A PRISMA-style flow summary is provided in Figure 1 to document identification, screening, eligibility, and inclusion decisions.

### 3.2. Study Characteristics

Table 2 summarizes the 10 articles that met the inclusion criteria and were published between 2018 and 2023. The study designs included five case reports, three longitudinal studies, and two prospective studies (evidence base skewed toward lower-level designs, limiting causal inference and generalizability). The age range of participants in these studies was 15–71 years, with the majority between 15 and 35 years old. The diagnostic tools or methods for assessing TMD included various radiographs (cephalometric, panoramic, computed tomography, cone-beam computed tomography, magnetic resonance imaging), clinical examinations, occlusal analysis, joint vibration analysis, and questionnaires. Radiographs were the most frequently used modality, applied alone in four studies [32,33,34,35] and combined with clinical examination in three studies [36,37,38]. The types of TMD examined included myofascial pain or myalgia, disk displacement, and osteoarthritis, with other types such as idiopathic condylar resorption and combinations of multiple TMD types also represented. One article did not specify the exact TMD subtype [36]. The outcome of each study is summarized in Table 2, detailing treatment specifics when mentioned and the effect of orthodontics on TMD symptoms. Given heterogeneity in design, diagnostics, and outcomes, quantitative pooling was not undertaken; results are synthesized narratively.

### 3.3. Orthodontic-TMD Relationship Findings

The outcomes depicted in Table 2 were categorized based on the treatment rendered. The first category included various traditional non-surgical orthodontic therapies, such as fixed appliance therapy, camouflage treatment, mini-implants, Herbst appliances, and stabilization splints [25,32,33,34,36,37,39]. These articles were further classified based on whether the treatment resulted in improvement or elimination of TMD symptoms or no effect on TMD. The second category focused on the surgical orthodontic approach, which included pre-orthodontic treatment, orthognathic surgery, and post-surgical orthodontic treatment [35,38,40]. The outcomes of this treatment were either improvement or elimination of TMD [38,40] or no effect on TMD [35]. The types of orthognathic surgery included in these studies were bilateral sagittal split osteotomy (BSSO) and LeFort I [35,38,40]. Briefly, BSSO repositions the distal mandibular segment relative to the proximal rami to correct sagittal and transverse discrepancies; Le Fort I repositions the maxilla to optimize occlusion and facial proportions. A detailed breakdown of the surgical and non-surgical orthodontic treatments and their outcomes can be seen in Table 3.

Non-surgical treatments (7/10 studies; 70%) included fixed appliance therapy, camouflage treatment, mini-implants, Herbst appliances, and stabilization splints [25,32,33,34,36,37,39]. Giray & Sadry [36] and Myllymäki et al. [39] found no association between orthodontic treatment and TMD symptom onset or burden while Hoshi et al. [37], Kaku et al. [32], Kato & Ono [33], and Lee et al. [34] described short-term improvements in pain and/or function. Ruf & Bock [25] observed symptom reduction immediately after treatment but no durable preventive effect at ≥15 years of follow-up. Consistent with Table 3, key outcome metrics included instrumented occlusal timing (e.g., disclusion time), joint vibration analysis, validated symptom questionnaires, RDC/DC-TMD classifications/indices, surface EMG, and clinician-reported pain/function and joint sounds. Overall, 4/7 studies showed short-term improvement, 2/7 showed no effect, and 1/7 showed mixed durability (early improvement with long-term attenuation) (Table 3).

Surgical approaches (3/10 studies; 30%) comprised staged care with pre-orthodontics, orthognathic surgery (BSSO and/or Le Fort I), and post-surgical orthodontics [35,38,40]. Kau et al. [38] and Paunonen et al. [40] reported improvements, particularly in pain prevalence and function, whereas Zibo et al. [35] found no clear short-term benefit. As summarized in Table 3, surgical studies commonly used DC/TMD Axis I diagnoses, maximum interincisal opening, palpation pain/muscle tenderness, joint sounds, and symptom prevalence as primary metrics. Two of three studies (~67%) suggested improvement, while one (~33%) showed no effect in the short term; duration beyond 12–24 months remains inconsistently assessed.

## 4. Discussion

### 4.1. Principle Findings and Context

Prior studies have explored the role of orthodontic interventions in alleviating TMD symptoms with varying results. These inconsistent results have led to ongoing debate among orthodontists and other dental experts about the most effective treatment approaches for TMD. Consistent with our Results, the studies examined in this review suggest that some orthodontic treatment modalities can improve or eliminate TMD symptoms [38,40], whereas others show no measurable benefit. A notable supplemental orthodontic intervention used in these studies is the use of mini-screws [32,33,34,37,38], also known as temporary anchorage devices (TADs). These devices provide additional anchorage and facilitate complex tooth movement during orthodontic treatment [41]. During TMD management, mini-screws may be particularly effective in cases involving vertical discrepancies by providing stable anchorage points for orthodontic mechanics, thus aiding in the correction of malocclusions and potentially decreasing occlusal forces on the TMJ [37], which may contribute to symptom relief and improved jaw function. Framed within the contemporary biopsychosocial model of TMD, these signals should be interpreted cautiously: occlusion and joint loading may contribute for selected patients, but they are not singular causal agents, and irreversible treatments should not be described as curative.

### 4.2. Non-Surgical Orthodontic Interventions

Research conducted by Giray & Sadry [36] suggests a transient decrease in disocclusion time during orthodontic treatment without a concurrent increase in TMD symptoms. In contrast, Hoshi et al. [37] reported symptomatic improvement following orthodontic care, highlighting the variability of TMD presentations and the need for individualized treatment strategies. Across the seven non-surgical studies, four reported short-term improvement in pain and/or function, two reported no effect, and one reported mixed results characterized by early improvement with attenuation at longer follow-up (Ruf & Bock [25]). As detailed in Table 3, key outcome metrics included instrumented occlusal timing (e.g., disocclusion time), joint vibration analysis, surface EMG, clinician-reported pain/function and joint sounds, and validated symptom questionnaires—though standardized patient-reported outcomes (PROs) were infrequently used. The variability in outcomes observed among the studies may stem from several factors, including but not limited to differences in case definitions and diagnostic criteria for TMD, variations in orthodontic treatment modalities, and heterogeneity in patient characteristics (age, sex, parafunctional habits, psychosocial comorbidities). Each of these could contribute to the disparities in conclusions. For instance, studies that employed different methods for diagnosing TMD, such as clinical examination, various imaging protocols, or questionnaires, may show variations in results. Additionally, the complexity of TMD as a multifactorial condition underscores the need for tailored treatment approaches, which helps explain the discrepancies in the effectiveness of orthodontic interventions across the studies. Natural history effects (e.g., regression to the mean) and short follow-up intervals may further inflate apparent short-term benefits while obscuring long-term trajectories.

### 4.3. Surgical Orthodontic Interventions

Furthermore, the combined orthodontic–surgical approach has been proposed as a promising treatment modality for relieving TMD symptoms and improving oral health outcomes in selected patients [37]. This method addresses skeletal discrepancies and malocclusions through orthognathic surgery in conjunction with various orthodontic treatments to alleviate TMD symptoms and improve function for some individuals. Studies emphasized the importance of utilizing orthodontic and orthognathic surgical methods to achieve stable occlusion, facial harmony, and potentially long-lasting relief from TMD symptoms [38,40]. However, surgical correction may also alter condylar loading, with uncertain effects in patients at risk for degenerative joint change; careful case selection and close postoperative monitoring are therefore warranted. Paunonen et al. [40] focused on the combined orthodontic–surgical approach and concluded that this approach improves TMD symptoms [38,40], highlighting the potential benefits of integrating surgical interventions with orthodontic treatment. However, Zibo et al. [35] found that an orthodontic–surgical approach did not lead to significant improvements in TMD symptoms, indicating the need for further investigation and standardized treatment protocols. Taken together, two of three surgical studies (~67%) reported improvement, particularly for pain prevalence, while one (~33%) showed no short-term benefit; durability beyond 12–24 months remains inconsistently assessed.

### 4.4. Outcomes by Domain (Pain, Function, Joint Sounds, PROs)

Pain. Short-term pain reduction is frequently observed in case-based non-surgical reports, and in one comparative surgical cohort pain-related diagnoses were less prevalent after mandibular advancement [40]. However, longer-term or higher-level evidence does not show a consistent preventive effect of orthodontic treatment on TMD; notably, Ruf & Bock [25] reported early improvement followed by attenuation at ≥15 years’ recall.

Function (e.g., mouth opening, masticatory performance). Several reports described improved function (e.g., increased maximum interincisal opening after surgical revision; improved jaw-movement symmetry with non-surgical care). Still, objective functional metrics were not consistently captured or harmonized across studies, limiting comparability.

Joint sounds. Changes in clicking/crepitus were variably reported and inconsistently linked to symptoms. Given the weak correlation with pain and function, joint sounds should be interpreted as secondary outcomes tied to standardized diagnostics.

PROs/quality of life. Validated PROs were rarely used across the corpus, constraining patient-centered interpretation. Future work should incorporate validated measures of pain interference, oral health–related quality of life, sleep disturbance, and treatment burden.

### 4.5. Diagnostics and Applicability

Overall, the diagnostic approaches for TMD analyzed in the reviewed articles encompass a variety of methods, highlighting the diverse strategies employed in assessing this condition. The methods include various radiographic techniques, clinical examinations, occlusal analysis, joint vibration analysis, and questionnaires. Radiographs emerged as the most commonly used diagnostic tool, employed alone in four studies and combined with clinical examinations in three others. Notably, only the study by Ruf & Bock [25] incorporated the Research Diagnostic Criteria for TMD (RDC/TMD), developed by Dworkin, and few studies referenced the updated DC/TMD framework—criteria widely regarded for enhanced reliability and validity [42]. Imaging frequently served as a primary diagnostic tool in the included literature, which is misaligned with best practice wherein imaging is adjunctive to a thorough history and clinical examination. Routine adoption of DC/TMD, standardized indications for imaging, and consistent use of validated PROs would materially improve interpretability and cross-study comparability.

### 4.6. Risk of Bias, Heterogeneity, and Durability

The evidence base is dominated by case reports and small observational studies, introducing selection bias, confounding by indication, regression to the mean, and potential publication bias. Heterogeneous diagnostics and outcomes further reduce certainty and preclude causal inference. Duration is insufficiently assessed across the studies; where longer follow-up exists, apparent benefits often attenuate (e.g., Ruf & Bock [25]). These factors collectively argue for cautious interpretation and conservative clinical translation of short-term improvements.

### 4.7. Limitations and the Evidence of This Review

This study has several limitations that should be acknowledged. One major limitation is the inclusion of a variety of TMD types, often in combination with more than one type. This diversity in TMD types can introduce significant heterogeneity in the results, making it challenging to draw consistent and specific conclusions about the impact of orthodontic treatment on TMD. The variability in TMD presentation can lead to difficulties in standardizing the outcomes and comparing results across different studies.

Another limitation is the lack of specification regarding the type of TMD assessed in some of the articles reviewed. One study did not clearly differentiate between the various subtypes of TMD, such as myofascial pain, disk displacement, or degenerative joint disorders. This lack of specificity can obscure the understanding of which particular forms of TMD respond best to orthodontic interventions. Consequently, this can contribute to inconsistent generalizations about the findings and reduce the overall clarity and applicability of the results.

Furthermore, the inclusion criteria for the studies might have led to a selection bias, potentially affecting the generalizability of the findings. Studies that were excluded due to language barriers, publication status, or availability of full text could have provided additional insights or different perspectives on the relationship between orthodontic treatment and TMD. In addition, our U.S.-only inclusion maximizes applicability to U.S. practice patterns but may limit international generalizability, a tradeoff we acknowledge explicitly. Finally, restricting the analytic window to 2018–2023 prioritizes contemporary modalities yet omits earlier foundational work; consequently, our interpretations should be viewed within the established biopsychosocial understanding of TMD and recognized as non-exhaustive of the historical literature.

### 4.8. Implications for Practice

Our findings have significant implications for patient care, underscoring the importance of considering the potential impact of orthodontic treatment on TMD symptoms. Clinicians should prioritize patient-centered outcomes, focusing on improvements in quality of life, effective pain management, and optimal jaw function when making treatment decisions. Additionally, engaging patients in shared decision-making is crucial. This involves thoroughly discussing the potential risks and benefits of orthodontic interventions for TMD, ensuring patients are well-informed about their treatment options. A practical, conservative-first pathway is appropriate: (1) confirm diagnosis using RDC/TMD or DC/TMD where applicable; (2) initiate conservative care (education, self-management, splints, physical therapy, behavioral strategies); (3) consider orthodontic mechanics, including TAD-supported mechanics, when malocclusion plausibly contributes to loading patterns; (4) reserve orthognathic surgery for clearly indicated skeletal discrepancies; and (5) monitor with validated PROs and functional metrics at defined intervals. Clinicians should avoid promising cure, emphasize uncertainty around long-term effects, and tailor plans to TMD subtype, comorbidities, and patient priorities.

### 4.9. Future Research

Future research should clarify TMD’s mechanisms and risk factors to establish evidence-based orthodontic management protocols [43]. Understanding how orthodontic treatments affect TMD can improve patient outcomes and quality of life. Key areas include studying the impact of occlusal adjustments, bite correction, and teeth realignment on TMD symptoms and force distribution within the TMJ [44]. Methodologically, future studies should be pre-registered, multi-center, and adequately powered; employ standardized diagnostics (RDC/TMD or DC/TMD); prespecify core outcome sets (pain intensity, jaw function, joint sounds, quality of life, adverse events); and ensure ≥12–24 months of follow-up to capture durability. Comparative-effectiveness trials of fixed appliances vs. clear aligners versus TAD-supported mechanics, with stratification by TMD subtype (myogenous versus arthrogenous) and sex, are particularly needed.

Identifying genetic predispositions and anatomical variations that influence treatment efficacy can help tailor personalized treatment plans [45]. Developing these protocols requires a multidisciplinary approach, incorporating robust clinical evidence and best practices [46]. Long-term studies are essential to assess the sustainability and durability of orthodontic treatment outcomes for TMD management [47]. Linking biomechanical modeling and advanced imaging of load-transfer to clinical outcomes, and building pragmatic registries with cost-effectiveness analyses, will further inform real-world decision-making.

### 4.10. Conclusions

This study highlights the complex interplay between orthodontic treatment and TMD. While certain studies demonstrate that both orthodontic treatment and the orthodontic–surgical approach yield positive results in reducing TMD symptoms, many studies have found no significant relationship between orthodontics and the development or worsening of TMD. Accordingly, clinicians should favor conservative first-line care, consider orthodontics when malocclusion plausibly contributes to joint loading or function, and reserve surgery for clearly indicated skeletal problems, acknowledging that long-term symptom resolution is not guaranteed. Overall, evidence remains heterogeneous and often short-term; standardized diagnostics, longer follow-up, and rigorous comparative-effectiveness research are necessary to identify prognostic factors and the patient subgroups most likely to benefit.

## Figures and Tables

**Table 1:** Inclusion and exclusion criteria for article selection.

Inclusion Criteria	Exclusion Criteria
Research articles between 2018 and 2023 Published peer-reviewed articles Human-based studies only in an orthodontic context Deals with treatment and or cause of TMD English language U.S. setting	Full text not available Review Articles (Narrative, Scoping, Systematic, Meta-Analyses), Opinions, Editorials Unpublished or retracted articles. Preprints Multinational studies without separable U.S. data No TMD outcomes reported

**Table 2:** Summary of basic characteristics of the study.

Author (Year)	Types of TMD	Study Design	Diagnostic Method(s)	Key Outcome
Giray & Sadry (2021) [36]	Not specified	Prospective	CE, Lat ceph, Pan, JVA, occlusion analysis	No association with TMD development
Hoshi et al. (2022) [37]	MP, DD	Case Report	CE, MRI, CT, interview	Ortho + mini-implants → pain resolved; no recurrence at 2 years
Kaku et al. (2019) [32]	OA, DDwR	Case Report	MRI	Ortho + stabilization splint → pain resolved during treatment
Kato & Ono (2018) [33]	OA	Case Report	EMG, CBCT	Pain resolved; no recurrence at 2 years
Kau et al. (2020) [38]	TMD (significant pain), ICR	Case Report	CBCT, full ortho records, clinical photos	Ortho + orthognathic surgery → symptoms eliminate
Lee et al. (2019) [34]	ICR, TMD	Case Report	CBCT, Lat ceph, Pan	Ortho + mini-implants + stabilization splint → symptoms resolved; no recurrence at 2 years
Myllymäki et al. (2023) [39]	MP, arthralgia, TMJ clicking	Prospective	Questionnaire, CE	No association between orthodontic treatment and TMD symptoms
Paunonen et al. (2019) [40]	Myalgia, MP, arthralgia, DJD, DDWR, DDWoR	Longitudinal	Questionnaire, CE	Ortho + orthognathic surgery ↓ TMD prevalence
Ruf & Bock (2019) [25]	Myalgia, Arthralgia, DJD, DD	Longitudinal	CE	Herbst-based orthodontics: ↓ symptoms post-treatment; no long-term effect
Zibo et al. (2022) [35]	Myalgia, arthralgia	Longitudinal	Cephalometric radiographs	Ortho + orthognathic surgery: no effect on prevalence/severity

MP = myofascial pain; DD = disk displacement; DDwR = with reduction; DDwoR = without reduction; OA = osteoarthritis; DJD = degenerative joint disease; ICR = idiopathic condylar resorption; CE = clinical examination; Lat ceph = lateral cephalogram; Pan = panoramic radiograph; JVA = joint vibration analysis; EMG = electromyography; CBCT = cone-beam CT.

**Table 3:** Outcomes of orthodontic interventions for TMD.

Author (Year)	Intervention	Key Outcome Metrics Used	Duration/Follow-Up	Overall Signal
Giray & Sadry (2021) [36]	Non-surgical	T-Scan^®^ occlusal parameters (e.g., disclusion time), chewing pattern analysis; Joint Vibration Analysis (JVA) for TMD screening	During active treatment (~6 months)	No effect on TMD onset/progression
Myllymäki et al. (2023) [39]	Non-surgical	Self-reported TMD symptoms questionnaire at ages 12/15/32; PAR Index (occlusal severity/changes); multivariable logistic regression (OR, 95% CI) (e.g., TMJ sounds, headache)	Longitudinal (~20 years)	No effect (treatment not a driver); risk factors noted
Ruf & Bock (2019) [25]	Non-surgical	RDC/TMD & DC/TMD clinical classification; Helkimo Index; prevalence of signs/symptoms across timepoints	Long-term (≥15 years)	Mixed/no durable effect
Hoshi et al. (2022) [37]	Non-surgical	6-DOF jaw-movement recording (Gnathohexagraph: incisal/condylar path lengths; symmetry); TMJ MRI (disk position); cephalometrics; clinical TMJ sounds & pain	~24 months (with extended retention notes)	Improved
Kato & Ono (2018) [33]	Non-surgical	Surface EMG of masticatory muscles (masseter/temporalis/digastric) during MVC; CBCT of TMJ; cephalometrics; clinical symptom status	~2–2.5 years	Improved
Kaku et al. (2019) [32]	Non-surgical	TMJ MRI (disk displacement), cephalometric angles (ANB, FMA, etc.); occlusal relationships; clinical TMD symptoms; panoramic/Schüller radiographs	~2 years	Improved
Lee et al. (2019) [34]	Non-surgical	Clinical joint symptom status (pain/clicking); occlusal stability; routine orthodontic records/radiographs (case-level reporting)	~2 years	Improved
Paunonen et al. (2019) [40]	Surgical	DC/TMD Axis I subdiagnoses (e.g., myalgia, arthralgia); symptom questionnaire; comparison of prevalence vs. non-treated Class II controls	Medium term (~4–8 years)	Improved (pain prevalence)
Zibo et al. (2022) [35]	Surgical	Mandibular movement amplitude (incl. mouth opening and deviation), TMJ pain, pathological TMJ sounds, masticatory muscle tenderness on palpation; cephalometrics, overjet/overbite	Short-term (14 days & 6 months)	No clear benefit (short-term)
Kau et al. (2020) [38]	Surgical	Maximum interincisal opening (MIO), lateral excursions, subjective pain/dysfunction, airway dimension (post-op), occlusal stability	Short-term (months)	Improved

## Data Availability

No new data were created or analyzed in this study.

## Supplementary Materials

The following supporting information can be downloaded at: https://www.mdpi.com/article/10.3390/clinpract15100182/s1, Table S1. Databases and Search Strategies.
