# Supplementary material for: Treating Temporomandibular Disorders Through Orthodontics: A Scoping Review of Evidence, Gaps, and Clinical Guidance
Source: Clin Pract. 2025 Sep 30;15(10):182. doi: 10.3390/clinpract15100182 (PMC12562917; doi:10.3390/clinpract15100182)
Supplement: Supplementary file 1 [file clinpract-15-00182-s001.zip › clinpract-3851417-supplementary.pdf]

Table S1. Databases and Search Strategies.

Databases	Search Strategy & Filter	Number of Results
Pubmed<br><br>https://pubmed.ncbi.nlm.nih.gov/?otool=nvujrolib<br><br>09/13/2023	("Orthodontics"[Mesh] OR "Orthodontics, Corrective"[Mesh]) AND ("Temporomandibular Joint Disorders"[Mesh] OR "TMD") AND ("Malocclusion"[Mesh] OR "occlusion")<br><br>Filter: English, Human, & 2018-2023	40
Scopus<br><br>https://www.scopus.com/search/form.uri?display=basic#basic<br><br>09/13/2023	(TITLE-ABS-KEY ((("Orthodontics") OR ("Orthodontics, Corrective") OR ("Corrective Orthodontics")) AND (("Temporomandibular Joint Disorders") OR ("Disorder, Temporomandibular Joint") OR ("Disorders, Temporomandibular Joint") OR ("Joint Disorder, Temporomandibular") OR ("Joint Disorders, Temporomandibular") OR ("Temporomandibular Joint Disorder") OR ("TMJ Disorders") OR ("Disorder, TMJ") OR ("Disorders, TMJ") OR ("TMJ Disorder") OR ("Temporomandibular Disorders") OR ("Disorder, Temporomandibular") OR ("Disorders, Temporomandibular") OR ("Temporomandibular Disorder") OR ("Temporomandibular Joint Diseases") OR ("Disease, Temporomandibular Joint") OR ("Diseases, Temporomandibular Joint") OR ("Joint Disease, Temporomandibular") OR ("Joint Diseases, Temporomandibular") OR ("Temporomandibular Joint Disease") OR ("TMJ Diseases") OR ("Disease, TMJ") OR ("Diseases, TMJ") OR ("TMJ Disease") OR (tmd ")) AND ((" malocclusion ") OR (" malocclusions ") OR (" tooth AND crowding ") OR (" crowding, AND tooth ") OR (" crowdings, AND tooth ") OR (" crossbite ") OR (" crossbites ") OR (" cross AND bite ") OR (" bite, AND cross ") OR (" bites, AND cross ") OR (" cross AND bites ") OR (" angle's AND classification ") OR (" angle AND classification ") OR (" angles AND classification ") OR (" classification, AND angle's ") OR (" occlusion "))) AND ALL(((" men') OR ("women") OR ("patient") OR ("female") OR ("male") OR ("subjects") OR ("adult") OR ("human"))) AND NOT ALL (("animal models"))) AND PUBYEAR > 2017 AND PUBYEAR < 2024 AND (LIMIT-TO (EXACTKEYWORD , "Human") OR LIMIT-TO (EXACTKEYWORD , "Humans")) AND (LIMIT-TO (LANGUAGE , "English")) AND (LIMIT-TO (AFFILCOUNTRY , "United States"))<br><br>Filters: Human, English, United States, & 2018-2023	625
Web of Science<br><br>https://www.webofscience.com/wos/woscc/basic-search<br><br>09/13/2023	(((TS=(("Orthodontics") OR ("Orthodontics, Corrective") OR ("Corrective Orthodontics")))) AND TS=(("Temporomandibular Joint Disorders") OR ("Disorder, Temporomandibular Joint") OR ("Disorders, Temporomandibular Joint") OR ("Joint Disorder, Temporomandibular") OR ("Joint Disorders, Temporomandibular") OR ("Temporomandibular Joint Disorder") OR ("TMJ Disorders") OR ("Disorder, TMJ") OR ("Disorders, TMJ") OR ("TMJ Disorder") OR ("Temporomandibular Disorders") OR ("Disorder, Temporomandibular") OR ("Disorders, Temporomandibular") OR ("Temporomandibular Disorder") OR ("Temporomandibular Joint Diseases") OR ("Disease, Temporomandibular Joint") OR ("Diseases,	234

	Temporomandibular Joint") OR ("Joint Disease, Temporomandibular") OR ("Joint Diseases, Temporomandibular") OR ("Temporomandibular Joint Disease") OR ("TMJ Diseases") OR ("Disease, TMJ") OR ("Diseases, TMJ") OR ("TMJ Disease") OR (tmd ")) AND ((" malocclusion ") OR (" malocclusions ") OR (" tooth AND crowding ") OR (" crowding, AND tooth ") OR (" crowdings, AND tooth ") OR (" crossbite ") OR (" crossbites ") OR (" cross AND bite ") OR (" bite, AND cross ") OR (" bites, AND cross ") OR (" cross AND bites ") OR (" angle's AND classification ") OR (" angle AND classification ") OR (" angles AND classification ") OR (" classification, AND angle's ") OR (" occlusion "))) AND ALL=((" men') OR ("women") OR ("patient") OR ("female") OR ("male") OR ("subjects") OR ("adult") OR ("human"))) NOT ALL=(("animal models"))<br><br>Filters: Human, English, United States, & 2018-2023
